# Supplementary material for: A subset of ipRGCs regulates both maturation of the circadian clock and segregation of retinogeniculate projections in mice
Source: eLife. 2017 Jun 15;6:e22861. doi: 10.7554/eLife.22861 (PMC5513697; doi:10.7554/eLife.22861)
Supplement: Supplementary file 1. — DOI: http://dx.doi.org/10.7554/eLife.22861.017 [file elife-22861-supp1.docx]

| **Mouse lines/conditions** | **Strain** | **M1 ipRGCs** | **Non-M1 ipRGCs** | **Circadian behavior** | **Eye-specific segregation and visual acuity** |
| --- | --- | --- | --- | --- | --- |
| LD and dark reared | B6J |  |  | Dark reared: lengthened circadian period until exposed to light  LD reared: normal circadian period length | Normal eye-specific axonal segregation and visual acuity (cite for dark reared) |
| Enucleated | B6J |  |  | P0 enucleated: Do not photoentrain and free run with a lengthen circadian period  P60 enucleated: Do not photoentrain and free run with a normal circadian period | All retinal projections are removed. |
| *Opn4^Cre/+^* | B6J /129 hybrid | Express Cre | Express Cre | Normal circadian photoentrainment | Normal eye-specific axonal segregation and visual acuity |
| *Opn4^Cre/+^; Z/AP* | B6J /129 hybrid | Labeled with AP | Labeled with AP | Normal circadian photoentrainment | Normal eye-specific axonal segregation and visual acuity |
| *Opn4^LacZ/LacZ^* (MKO) | B6J /129 hybrid | Lack the melanopsin protein; labeled with x-gal | Lack the melanopsin protein | Normal circadian photoentrainment; small deficits in phase shifting | Normal eye-specific axonal segregation and visual acuity |
| *Opn4^aDTA/aDTA^* | B6J /129 hybrid | Ablated during adulthood; lack melanopsin | Persist but lack melanopsin | Do not photoentrain and free run with a normal circadian period | Normal eye-specific axonal segregation and visual acuity |
| *Opn4^aDTA/LacZ^* | B6J /129 hybrid | All but ~125 ablated during adulthood; remaining cells lack melanopsin; labeled with x-gal | Persist but lack melanopsin |  |  |
| *Opn4^Cre/aDTA^; Z/AP* | B6J /129 hybrid | All but ~125 ablated during adulthood; remaining cells lack melanopsin; labeled with AP | Persist but lack melanopsin; labeled with AP |  |  |
| *Opn4^DTA/+^* | B6J | All but ~75 are ablated by P14 | Many are ablated by P14 | Normal circadian photoentrainment | Intermediate deficits in eye-specific axonal segregation and visual acuity |
| *Opn4^DTA/LacZ^* | B6J | All but ~75 are ablated; remaining cells lack melanopsin; labeled with x-gal | Many are ablated by P14; remaining cells lack melanopsin | Highly disrupted photoentrainment |  |
| *Opn4^Cre/DTA^; Z/AP* | B6J | All but ~75 are ablated by P14; remaining cells lack melanopsin; labeled with AP | Many are ablated by P14; remaining cells lack melanopsin; labeled with AP |  |  |
| *Opn4^DTA/DTA^* | B6J | Ablated during postnatal development | Cannot be determined for sure, but many, if not all, are ablated during postnatal development; remaining cells lack melanopsin | Do not photoentrain and free run with a lengthen circadian period | Severe deficits in eye-specific axonal segregation and visual acuity |
| *Opn4^Cre/+^; Pou4f2^zDTA/+^*  (Previously published as  *Opn4^Cre/+^; Brn3b^DTA/+^*) | B6J /129 hybrid | All but ~200 ablated by P7 | Ablated by P7 | Normal circadian photoentrainment | Normal eye-specific axonal segregation and visual acuity |
| *Opn4^Cre/+^; Pou4f2^zDTA/^; Z/AP*  (Previously published as  *Opn4^Cre/+^; Brn3b^DTA/+^*; *Z/AP*) | B6J /129 hybrid | All but ~200 ablated by P7; labeled with AP | Ablated by P7; labeled with AP |  |  |
