# Supplementary material for: A subset of ipRGCs regulates both maturation of the circadian clock and segregation of retinogeniculate projections in mice
Source: eLife. 2017 Jun 15;6:e22861. doi: 10.7554/eLife.22861 (PMC5513697; doi:10.7554/eLife.22861)
Supplement: Supplementary file 2. — Properties of spontaneous retinal activity in P6 WT and Opn4DTA/DTA mice. Recordings were done in darkness. * represent statistically significant differences after Student's T-tests with a Holm-Bonferroni correction, α = 0.05, m = 22. Significance is determined when the p-value is less than or equal to the Holm-Bonferroni corrected p-value. DOI: http://dx.doi.org/10.7554/eLife.22861.018 [file elife-22861-supp2.docx]

| **Property** | **Ctrl Mean** |  | **Ctrl SEM** | **DTA Mean** |  | **DTA SEM** | **p value** | **Holm-Bonferroni** |
| --- | --- | --- | --- | --- | --- | --- | --- | --- |
| *** Number of Spikes** | 833.291 | ± | 43.442 | 1936.755 | ± | 134.387 | 0.000 | 0.003 |
| **Firing Rate Outside All Bursts** | 2.584 | ± | 0.315 | 4.429 | ± | 4.429 | 0.042 | 0.005 |
| **Firing Rate Outside WABs** | 6.647 | ± | 0.805 | 9.141 | ± | 1.345 | 0.112 | 0.007 |
| **ISI Outside All Bursts** | 73.485 | ± | 5.588 | 52.454 | ± | 6.399 | 0.014 | 0.004 |
| **ISI Outside WABs** | 51.017 | ± | 5.198 | 38.279 | ± | 4.762 | 0.071 | 0.006 |
| *** Percent of Spikes Outside All Bursts** | 6.802 | ± | 0.866 | 16.082 | ± | 1.979 | 0.000 | 0.003 |
| **Percent of Spikes Outside WABs** | 13.095 | ± | 1.333 | 19.926 | ± | 2.070 | 0.006 | 0.004 |
| **Burst Frequency** | 1.025 | ± | 0.025 | 1.016 | ± | 0.040 | 0.849 | 0.025 |
| *** Interburst Interval** | 60.472 | ± | 1.619 | 52.265 | ± | 2.085 | 0.002 | 0.003 |
| **Percent of Bursts That Are WABs** | 89.284 | ± | 1.149 | 90.945 | ± | 1.101 | 0.297 | 0.010 |
| **Number of WABs** | 9.313 | ± | 0.187 | 9.924 | ± | 0.266 | 0.061 | 0.005 |
| *** WAB Duration** | 3.376 | ± | 0.062 | 3.907 | ± | 0.078 | 0.000 | 0.003 |
| *** WAB Firing Rate** | 20.871 | ± | 0.851 | 41.741 | ± | 2.223 | 0.000 | 0.002 |
| *** WAB ISI** | 0.075 | ± | 0.003 | 0.054 | ± | 0.004 | 0.000 | 0.003 |
| *** Percent WAB Time >10 Hz** | 46.976 | ± | 1.396 | 57.992 | ± | 1.604 | 0.000 | 0.003 |
| *** Spikes Per WAB** | 79.753 | ± | 4.049 | 184.171 | ± | 10.832 | 0.000 | 0.002 |
| **Number of NonWABs** | 3.109 | ± | 0.390 | 4.071 | ± | 0.723 | 0.244 | 0.008 |
| **NonWAB Duration** | 2.270 | ± | 0.091 | 2.135 | ± | 0.133 | 0.402 | 0.013 |
| **NonWAB Firing Rate** | 11.896 | ± | 0.898 | 17.048 | ± | 2.206 | 0.033 | 0.004 |
| **NonWAB ISI** | 0.133 | ± | 0.008 | 0.130 | ± | 0.010 | 0.752 | 0.017 |
| **Percent NonWAB Time >10 Hz** | 28.478 | ± | 2.380 | 28.552 | ± | 3.057 | 0.985 | 0.050 |
| **Spikes Per NonWAB** | 30.641 | ± | 3.233 | 45.760 | ± | 7.748 | 0.074 | 0.006 |

= non-WAB

= Entire Recording

= WAB

Supplemental File 2: Values and statistics for retinal wave recordings
